# On the Operative Method for the Permanent Cure of Aernia

**Published:** 1855-04

**Authors:** 


### *On the Operative Method for the Permanent Cure of Hernia.*
By J. F. Malgaigne.

The various operations which have been devised for the radical cure of inguinal hernia may be included in two categories: those invented prior to the commencement of this century, all of which have reference to the external ring; and those of more recent date, in which, at last, the existence of an inguinal canal seems to be recognized.

*Castration.*—The first method which suggested itself to the empirics who, in old times, monopolized the treatment of hernia was extremely simple; there was an hernia in the scrotum: well! they cut off the scrotum and the testicle too. This procedure was expeditious, certainly, but its results were very far from being uniformly successful. It was a very popular method, and even after the invention of trusses, thousands were subjected to it. It was generally employed, not only for the cure but the prevention of hernia, until the commencement of the seventeenth century; but about this time, Fabricious of Aquapendente relates that trusses were used more extensively, and that Horace of Nortia, a famous castrator of the day, complained that his profession was ruined, for he then only removed fifteen or twenty wretched testicles a year, instead of two or three hundred, which had formerly been his annual harvest.

In 1840, there were still some itinerant charlatans who practiced this barbarous operation in the departments of France.

Richter says that German physicians of his day cured hernia by this method, and that the loss of testicle is not so great a price to pay for the relief of such an infirmity. It is probable he did not have an hernia to be operated on, else he would not have expressed himself so lightly.

No doubt this mutilation, irrational and barbarous as it appears, procured some cures. The external ring, being no longer traversed by the cord, or stretched by the weight of the testicle, may be supposed to contract somewhat, and the male subject, submitted to this operation, enters into the female category, and becomes four times less liable to hernia than he was before. It is possible, too, that the cord in retracting, and carrying a bleeding surface into the inguinal canal, may become covered with plastic lymph, may contract adhesions, and may excite an inflammation, which, by propagation to the internal ring, may obliterate the neck. But the contraction of the external ring required a long period; the obliteration of the neck was an exceptional occurrence, and before either of them was accomplished the hernia usually returned.

*Ligature.*—Another method was called the *punctum aurum;* it consisted in laying bare the sac and cord, and encircling them with a golden wire, which was gradually tightened until it cut itself out. The patient was left with a testicle deprived of its vessels.

*Spanish Method.*—An itinerant Spaniard, whose name has not come down to us, attempted the radical cure of hernia by forcing the testicle into the abdomen, and sewing up the integuments below. According to some accounts, he incised the sac, and practiced the *punctum aurum*, after getting the testicle within the abdomen.

*Suture.*—In the seventeenth century, Ambroise Paré denounced the practice of ablation of the testicle, "that organ" (and he calls it by a very vulgar name) "which preserves peace in the household;" he devised the operation of dissecting the sac from the cord, and tying it at the external ring. Other surgeons preferred several stitches to a single ligature, and, after isolating the sac, they sewed up a portion of it with the continued suture. They called this the *royal suture*, because it left the patient the ability to increase the king's subjects. Cures were doubtless attained by this method; inflammation, however induced, may extend to the internal ring and cause its obliteration; it may also extend to the peritoneum, and it often did so in the cases in which the royal suture was practised.

*Petit's Operation.*—You know that J. L. Petit advised that in the operation of strangulated hernia the structure should be relieved without opening the sac; a practice resorted to by Monro, in more modern times by Key, and still warmly advocated by Mr. Luke, of the London Hospital. Petit proposed further, that after the intestine was reduced, the sac itself should be returned into the abdomen, where it would probably contract adhesions and constitute a barrier. The idea is quite plausible, but its execution almost entirely involves the development of peritonitis. I acted on it once. The idea occurred to me while operating on a woman with strangulated crural hernia; the patient succumbed, but not from the cause I anticipated; suppurative inflammation had extended to the iliac fossa. I shall never attempt the like again.

*Cauterization.*—After the destruction of the sac by ligature and the bistoury, there was nothing left but to extirpate it by actual and potential cauteries, and this was accordingly done. You will find a historical account of this method in the annals of the Academy of Surgery. It was condemned in very plain terms by that learned company.*

Very recently, M. Vallette, a surgeon of Lyons, has executed a new method of cauterization, in which, he asserts, all danger of injuring the spermatic vessels is avoided. A kind of slab of ebony is forcibly crowded into the inguinal canal, the skin of the scrotum being pushed before it; a needle is glided along its internal surface, and is made to emerge toward the superior portion of the inguinal canal, and its thread is passed through an aperture in a wooden disk which is applied internally. By tightening the thread the slab and disk are brought into close apposition. They include between them the anterior wall of the disk, in which Vienna paste is put. The soft parts between the two slabs, so far as they are left uncovered by the external slab, are consequently destroyed. An eschar is produced involving the whole anterior wall of the canal, which is replaced by a firm cicatrix. During this

---

* The author refers to a report by Bordenave, in which the dangers of the operation of cauterization are fully exposed. See *les Annales de l'Academie royale de Chirurgie*, T. v., p. 651. *Translator's Note.*

process, M. Vallette's own experience proves that it is not possible to limit the action of caustic by his method, and that notwithstanding the frightful wound it produces and the dangers it involves it is not uniformly followed by success. No other surgeon, that I know of, has practiced M. Vallette's operation, which, it must be admitted, has little to recommend it.

*Belmas' Method.*—A plan for the cure of hernia devised by M. Belmas is exceedingly odd and strange. It is a method which makes one wonder how its author ever came to think of it,—something unique in pathological physiology. When the sac of a scrotal hernia has not formed adhesions, it may readily be pushed up to the external ring. In M. Belmas' method, then, the hernia was reduced, the sac pushed up, and a slight incision was made in the skin of the scrotum, through which a canula was introduced, and gently insinuated between the cord and adjacent parts until it reached the external ring. A long needle was passed through the canula, and brought out at the external ring; the thread of this needle was attached to a little pouch of gold beater's skin. This pouch was drawn up to the ring, and by means of a small tube it was expanded with air. Thus a plug was placed at the external ring. The subsequent results were very curious. Plastic lymph was thrown out in consequence of the presence of this foreign body, and this lymph passed through the gold beater's skin, in accordance with the laws of endosmosis, and became organized within and without the pouch. Thus an organized plug was placed at the outlet of the inguinal canal. M. Belmas practiced his operation repeatedly on dogs, for hernia is not uncommon in these animals, and his results were often successful. He tried it once on the human subject. The patient died of peritonitis, and the experiment has not been repeated. M. Belmas has told me that his idea was entirely empirical. It is evident, indeed, that none of the methods for the cure of hernia could have suggested such a singular contrivance.

*Bonnet's Method.*—A much simpler operation, recommended by M. Bonnet, of Lyons, is performed as follows: A pin, passed through a piece of pasteboard or cork, is introduced through the integuments, passed behind the neck of the sac, and brought out through the skin; another pin is passed in front of the sac; and a third behind it. Other pieces of cork are placed on the points of these pins; by approximating the corks, the opposite sides of the sac are brought into close apposition. The pins are left in place for about six days. This operation was proposed as a novelty, but it is in fact only a modification of the old royal suture, in which the skin is respected, and the chances of phlegmonous inflammation consequently lessened. There is a likelihood of obtaining adhesion of the middle portion of the sac by this operation; peritonitis, also, is to be apprehended.

*Jameson's Method.*—Jameson, a surgeon of Baltimore, performed an operation for the radical cure of femoral hernia, which consisted in cutting a lance-shaped flap of integuments, inverting it, and confining it by stitches in the crural ring, which had previously been exposed by the incisions required to relieve a strangulation. His operation is said to have been successful but he did not adopt it in other instances, or apply it to inguinal hernia. It occurred to Professor Gerdy to attempt this, but in carrying out his idea he modified Jameson's operation in so many respects as to be justly entitled to the credit of inventing a new method.

*Gerdy's Method.*—Even in fat subjects the finger may be carried, pushing the skin of the scrotum before it, to the external inguinal ring or a little beyond it. M. Gerdy reasoned that if the integuments could be maintained in this position, they would effectually plug up the ring and prevent protrusion. He devised the following operation. The finger, carrying before it the skin of the scrotum, is forced as far as possible into the inguinal canal. A long curved needle is passed to the extremity of the glove-finger or cul-de-sac thus formed; it is glided over the end of the finger which pushes up the skin, thrust through the integuments, and brought out about the middle of the inguinal canal. The free extremity of the thread carried by this first needle is now inserted into a second needle, which is passed in like manner through the extremity of the cul-de-sac and out through the inguinal canal, only the second needle is made to penetrate at a little distance from the first puncture. By pulling on the two ends of this thread the extremity of the plug formed by the invaginated skin of the scrotum is drawn as far as possible into the inguinal canal. It is fixed in this position by tying the thread around a cylinder of linen or diachylon, as in the quilled suture. The wall of the cul-de-sac is then denuded of its cuticle by the application of caustic ammonia. There are two chances of cure: by adhesion of the cul de-sac with the inguinal canal and peritonæum, and by adhesion of the wall of the pouch itself, and the consequent formation of a solid plug.

M. Gerdy practiced this operation on a number of patients, several of whom I saw. In some, the anticipated results seem to have been attained. There were adhesions externally, but the plug which ought to have obliterated the inguinal ring could not be felt. In these cases, Professor Gerdy did not dare to remove the bandage. I saw other cases which appeared to be entirely cured after the lapse of several months. In some instances, the invaginated skin resumed its former position in consequence of its weight and elasticity; in others, the hernia protruded as soon as a truss was left off. At that time, M. Gerdy complained that he had not cases enough to select those in which his method was applicable. He said he had only large herniæ to operate on. Inflammation ran high after some of his operations, and it was necessary to use cold water by the method of irrigation. One patient died of pleurisy in consequence of this treatment, and since this fatal event the operation has not been practiced.

I do not think it possible to plug the inguinal canal by M. Gerdy's method. The subject on the table has a large inguinal ring, yet if you endeavor to invaginate the skin of the scrotum, you will find it impossible to force it more than a quarter of an inch into the canal. In the patients cured by this method, it was not possible to feel any plug. There was, therefore, another element in the operation sufficient to effect a cure, and this was the suture, carried through the inguinal canal.

*Guérin's Method.*—It has been proposed to scarify the external wall of the sac after laying it bare. M. Jules Guérin has modified this idea by the application of the subcutaneous method. He introduces a tenotome into the inguinal canal, and attempts to scarify the tendon of the external oblique and also the sac, hoping to provoke adhesive inflammation between the aponeurosis and serous membrane. He also scarifies the external ring. The application here of the subcutaneous method is a happy idea, but the mode of scarifying advised by M. Guérin is not to be commended. Scarifications of the sac are highly dangerous, and the dilatation of the inguinal ring resulting from incisions made in it, removes every chance of cure.

*Malgaigne's Method.*—I, also, have had my little notion. I thought that by passing M. Bonnet's pins into the sac itself, there would be greater likelihood of producing obliteration. I passed several pins, and when I supposed sufficient inflammation to be induced, I applied a truss. It was impossible, however, to be certain that the pins were passed at the proper depth, and I have long since given up this operation.

None of the operations that I have described can be depended on for the radical cure of hernia. The method of Bonnet, however, deserves to be retained. In cases of direct inguinal hernia, and where the rings are greatly dilated, and it is impossible to keep up the hernia with a truss, slight adhesions produced by the compression of the pins will render it possible to use the truss more successfully.

In M. Gerdy's method, too, although the invaginated skin does not plug at all, the idea of passing a suture through the inguinal canal is most excellent, and I am convinced that if surgical science is ever enriched with a more successful operation for the radical cure of hernia, it will be by some modification of this suture.—*Virginia Med. and Surg. Journal.*

